# Experience and satisfaction of stroke patients with institutions in the network of care: the Portuguese insight

**Published:** 2012-09-04

**Authors:** Nina Szczygiel, Silvina Santana

**Affiliations:** Research Unit on Governance, Competitiveness and Public Policies, Department of Economics, Management and Industrial Engineering, University of Aveiro, Aveiro, Portugal; Institute of Electronics Engineering and Telematics of Aveiro, Department of Economics, Management and Industrial Engineering, University of Aveiro, Aveiro, Portugal

**Keywords:** patient satisfaction, service quality, network of care, health and social care systems, stroke, Portugal

## Abstract

**Purpose:**

To identify which institutions play an active role in health and social care delivery for stroke patients in the Portuguese setting and to determine the level of patient satisfaction from interactions with them.

**Theory:**

It has been generally acknowledged that patients' reports of their health and their satisfaction with care services are as important as many clinical health measures. Many players in the health care arena are nowadays using information on satisfaction when making decisions. The environment in which proper health care organizations are operating has been turning more and more competitive, and patient satisfaction has become a factor with a strong impact on maintaining their market position. This perhaps does not apply until the same extent into the social care organizations, which in Portugal often operate on a non-profit or charity basis.

**Methods:**

A randomized controlled trial on patients diagnosed with stroke and admitted to a hospital in Center Region, Portugal. The patients included in the study (eligibility criteria defined previously) have been followed within the next six months and their experiences and satisfaction with case-to-case specific entities involved in health and social care delivery have been evaluated.

**Results and conclusions:**

Our findings indicate that the stroke patient’s network of care has been constituted by a number of institutions, with hospital, health centre, physiotherapy centre (both public and private) and fire department (transportation services) being the key ones. The satisfaction level from the interactions has been relatively high for all these main care network members, with the highest scores for firemen and the acute care hospital. Also other entities, such as rehabilitation units or charity institutions, make part of this care setting, however, with a lesser frequency of use by the patient.

A great challenge for the modern health and social care systems is also how to manage a certain network of care as a whole, instead of considering solely issues related to its individual members.

**Discussion:**

There are several entities taking part in the stroke network of care, the subject of this study, all of them heading for providing quality care and satisfying patients’ needs while bearing in mind budget limitations. As literature suggests, the level of patient satisfaction will result from a comparison on what the patient expected from the service and how the delivered service has been perceived. The relation between satisfaction and service quality and its direction has been since long discussed in the literature. We believe that a level of patient satisfaction is derived from the quality of experienced service, and measuring the satisfaction can be subsequently used as a premise for further quality improvement.

